# Effect of SGLT2 inhibitors on body composition, fluid status and renin–angiotensin–aldosterone system in type 2 diabetes: a prospective study using bioimpedance spectroscopy

**DOI:** 10.1186/s12933-019-0852-y

**Published:** 2019-04-05

**Authors:** Anja Schork, Janine Saynisch, Andreas Vosseler, Benjamin Assad Jaghutriz, Nils Heyne, Andreas Peter, Hans-Ulrich Häring, Norbert Stefan, Andreas Fritsche, Ferruh Artunc

**Affiliations:** 1 Department of Internal Medicine IV, Division of Endocrinology, Diabetology, Vascular Disease, Nephrology and Clinical Chemistry, University Hospital Tübingen, Otfried-Müller-Str.10, 72076 Tübingen, Germany; 2 Institute of Diabetes Research and Metabolic Diseases (IDM), Helmholtz Center Munich at the University of Tübingen, Otfried-Müller-Strasse 10, 72076 Tübingen, Germany; 3 German Center for Diabetes Research (DZD), Otfried-Müller-Strasse 10, 72076 Tübingen, Germany; 4 Department for Diagnostic Laboratory Medicine, Institute for Clinical Chemistry and Pathobiochemistry, Otfried-Müller-Strasse 10, 72076 Tübingen, Germany

**Keywords:** Diabetes mellitus, SGLT2 inhibitor, Body composition monitor, Bioimpedance sprectroscopy, Fluid status, Overhydration, Renin–angiotensin–aldosterone system

## Abstract

**Background:**

SGLT2-inhibitors are potent antihyperglycemic drugs for patients with type 2 diabetes and have been shown to reduce body weight. However, it is unclear which body compartments are reduced and to what extent.

**Methods:**

In this longitudinal observational study, we analyzed the body composition of 27 outpatients with type 2 diabetes mellitus during the first week and up to 6 months after initiation of treatment with SGLT2-inhibitors (n = 18 empagliflozin, n = 9 dapagliflozin) using bioimpedance spectroscopy (BCM, Fresenius). Fluid status of hypertensive patients taking medication with hydrochlorothiazide (n = 14) and healthy persons (n = 16) were analyzed for comparison.

**Results:**

At 6 months, HbA1c decreased by 0.8% (IQR 2.3; 0.4), body weight and BMI by 2.6 kg (1.5; 9.3) and 0.9 kg/m^2^ (0.4; 3.3), respectively. Bioimpedance spectroscopy revealed significant decrease in adipose tissue mass and fat tissue index while lean tissue parameters remained stable. Overhydration (OH) and extracellular water (ECW) decreased by − 0.5 L/1.73 m^2^ (− 0.1; − 0.9) and − 0.4 L/1.73 m^2^ (− 0.1; − 0.8) at day 3, respectively, and returned to the initial value after 3 and 6 months. Plasma renin activity increased by 2.1-fold (0.5; 3.6) at 1 month and returned to the initial level at month 3 and 6. Fluid status of patients with SGLT2 inhibitors after 6 months showed no difference from that of hypertensive patients taking hydrochlorothiazide or healthy persons.

**Conclusions:**

Body weight reduction under the treatment with SGLT2-inhibitors is caused by reduction of adipose tissue mass and transient loss of extracellular fluid, which is accompanied by upregulation of renin–angiotensin–aldosterone system (RAAS). Permanent loss of extracellular water does not occur under SGLT2 inhibition.

**Electronic supplementary material:**

The online version of this article (10.1186/s12933-019-0852-y) contains supplementary material, which is available to authorized users.

## Background

Inhibitors of the sodium-coupled glucose transporter 2 (SGLT2) are a promising and increasingly prescribed class of oral antidiabetic drugs. SGLT2-inhibitors increase urinary excretion of glucose by inhibiting its reabsorption via SGLT2 in the proximal tubule of the kidney, thus lowering blood glucose levels [1]. Besides their antidiabetic effect, loss of body weight has been observed consistently [2–4]. However, it is uncertain which body compartments and tissues change after the initiation of SGLT2 inhibition. In theory, body weight loss under treatment with SGLT2 inhibitors could be either due to loss of fat mass by a negative effect on the energy balance or due to loss of sodium and extracellular volume by a diuretic effect or due to a combination of both. Previous investigations of changes in body composition under SGLT2 inhibitors mainly relied on calculated indices or x-ray absorptiometry [5–7]. In contrast, quantitative measurements of body composition have only lately been performed in a few cohorts [8–11]. Early changes of body composition during the first days after initiation of SGTL2 inhibitors have not been investigated yet.

In addition to the antihyperglycemic effect, treatment with SGLT2 inhibitors was associated with reduction of hospitalization and mortality due to heart failure [12–16]. Up to now, the underlying mechanisms remain ill-defined, further studies investigating this connection are carried out [17] and different theories have been discussed [18–20]. Due to concomitant natriuresis of SGLT2 inhibitors, a direct diuretic effect with reduction of extracellular volume is conceivable. The early separation of the Kaplan–Meier curves for heart failure hospitalization and mortality in patients treated with empagliflozin [12] suggests an immediate effect on congestive symptoms and strikingly resembles that of thiazide diuretic chlorthalidone as observed in the ALLHAT Treatment Group in 2007 [21]. Bioimpedance spectroscopy analysis as a quantitative measurement method can contribute to further define changes of body composition under treatment with SGLT2 inhibitors and to identify changes in fluid status that could be responsible for these favorable effects in heart failure.

In this study, we analyzed the course of body composition and fluid status as measured by bioimpedance spectroscopy in a cohort of patients with type 2 diabetes after initiation of therapy with SGLT2 inhibitors empagliflozin or dapagliflozin during the first week and a follow up period of 6 months. Additionally, body composition and fluid status of patients with arterial hypertension and established diuretic therapy with hydrochlorothiazide, and control groups without SGLT2 inhibitors or diuretic medication were analyzed.

## Methods

### Patients

This study included patients with type 2 diabetes presenting for improvement of antidiabetic therapy at the Department of Internal Medicine of the University Hospital of Tübingen between March 2017 and June 2018. They all received standardized diabetes group training during 5 days which included dietary and lifestyle advice. Furthermore, the drug treatment (antidiabetic, antilipidemic and antihypertensive drug treatment) was adjusted daily to optimize according to the principles of the guidelines of the German Diabetes Association, the STENO Study [22] and the recently published consensus report of the American Diabetes Association and the European Association for the Study of Diabetes [23]. Patients with type 2 diabetes starting medication with a SGLT2 inhibitor due to indication were included after they provided written informed consent (n = 27). Patients were not included when they had changes in diuretic therapy, history of a clinical condition leading to changes in body composition like malignant tumor, aggressive diet regimen or bariatric surgery or evidence of liver disease, or declined to participate (n = 10).

To further compare the fluid status under SGLT2 inhibition with that of healthy individuals, patients with type 2 diabetes without SGLT2 inhibition and hypertensive patients, data obtained from a former study of our group, involving patients with chronic kidney disease of all stages and hypertension, [24] were re-assessed. To ensure comparability, patients with GFR > 60 mL/min/1.73 m^2^ and albuminuria < 30 mg/g creatinine were selected for the analyses of this manuscript.

### Evaluation of body composition and fluid status

Body composition and fluid status were assessed by bioimpedance spectroscopy using the Fresenius body composition monitor (BCM) which is optimized for dry weight estimation of dialysis patients [25]. Bioimpedance measurements performed at a spectrum of 50 frequencies between 5 and 1000 kHz enable to differentiate between intra- and extracellular fluid, as low electronic currents cannot pass cell membranes and flow through extracellular water only [26]. Parameters of volume status and body composition are calculated by the BCM using two physiological models: extracellular water (ECW), intracellular water (ICW) and total body water (TBW) are calculated using the body volume model; the body composition model differentiates normally hydrated fat mass, normally hydrated lean mass and a remaining proportion of water, and lays the foundation to calculate parameters of adipose tissue (adipose tissue mass, ATM and fat tissue index, FTI), lean tissue (lean tissue mass, LTM and lean tissue index, LIT) and the so-called overhydration (OH) [27]. OH is mainly part of extracellular fluid and reference values for OH lie between − 1 and + 1 L. Values obtained for OH, ECW and ICW were normalized to a body surface area of 1.73 m^2^.

### Laboratory assays

Blood and spot urine samples were drawn from each patient. Urine and plasma creatinine concentrations were determined using an enzymatic assay on the ADVIA XPT clinical chemical analyzer (Siemens Healthineers, Eschborn, Germany). Urine protein was determined using a turbidimetric benzethonium chloride assay (Roche Diagnostics, Mannheim, Germany) on the latter instrument. HbA1c measurements were performed using the Tosoh glycohemoglobin analyzer HLC-723G8 (Tosoh Bioscience Tokyo Japan). Serum aldosterone and plasma renin activity were measured manually using RIA methods (Immunotech, Prague, Czech Republic; Zentech, Angleur, Belgium).

### Statistical analysis

For parameters obtained at baseline and follow up measurements of patients with type 2 diabetes starting medication with a SGLT2 inhibitor, Friedman test was used to test for significant changes during the whole period of follow up. Friedman test only analyses data from patients with complete follow up and without missing values. Wilcoxon Signed-Rank test was used as post-test for differences between respective points of follow up. ANOVA and t-test for each pair as post-test was used to test for in between group differences of healthy controls, patients with type 2 diabetes with and without SGLT2 inhibitor and patients with hypertension with and without hydrochlorothiazide treatment. Bonferroni correction for multiple testing was performed. Data analysis was performed using the statistical software packages SAS Institute Inc. JMP 13.0, IBM SPSS Statistics 25.0 and GraphPad Prism 4.03.

## Results

### Characterization of the study cohort

The study included consecutively n = 27 patients with type 2 diabetes starting medication with an SGLT2 inhibitor (n = 1 empagliflozin 5 mg; n = 5 empagliflozin 10 mg; n = 12 empagliflozin 25 mg; n = 1 dapagliflozin 5 mg; n = 8 dapagliflozin 10 mg). Baseline characteristics of the study cohort are provided in Table 1. Median age was 58 years (interquartile range 52; 68), n = 12 (43%) patients were male and median BMI was 36.3 kg/m^2^ (32.6; 46.1) at baseline, respectively. All patients had normal renal function and almost no proteinuria. Altogether n = 11 patients completed all four follow-up visits. N = 17 patients were lost to follow up because they had stopped medication with the SGLT2 inhibitor (n = 2) or because they declined or were unable to participate in follow up (n = 15). Adverse events from medication with the SGTL2 inhibitor were reported by n = 3 patients (all urogenital infections). Disturbing polyuria especially during the first days of intake was reported by some patients.

**Table 1:** Baseline characteristics of patients before starting medication with a SGLT2 inhibitor

Variable	Value
Number of patients	27 Empagliflozin 18 (1 × 5 mg; 5 × 10 mg; 12 × 25 mg) Dapagliflozin 9 (1 × 5 mg; 8 × 10 mg)
Age (years)	58 (52; 68)
Sex (male)	12 (43%)
BMI (kg/m^2^)	36.3 (32.6; 46.1)
Body weight (kg)	101.9 (87.8; 128.6)
Blood pressure	
Systolic (mmHg)	141 (129; 153)
Diastolic (mmHg)	88 (76; 99)
Heart rate (1/min)	78 (70; 88)
Antidiabetic medication	
Insulin	20 (71.5%)
Metformin	26 (92.9%)
DPP4 inhibitor	12 (42.9%)
GLP1 agonist	7 (25%)
Therapy with RAAS inhibitor	25 (93%)
ACE inhibitor	20 (74%)
Angiotensin II receptor antagonist	5 (18%)
Statin therapy	15 (56%)
HbA1c (%)	9.1 (8.1; 10.9)
Creatinine (mg/dL)	0.7 (0.6; 0.9)
Proteinuria (mg/g creatinine)	124 (78; 185)
Hematocrit (%)	42 (39; 44)
Uric acid (mg/dL)	4.7 (4.3; 5.9)
Cholesterol (mg/dL)	172 (160; 200)
HDL cholesterol (mg/dL)	46 (37; 54)
LDL cholesterol (mg/dL)	104 (92; 135)
Macrovascular complications: history of	
Coronary artery disease/myocardial infarction	10 (37%)
Stroke	2 (7%)
Peripheral artery disease	1 (4%)
Microvascular complications: history of	
Diabetic retinopathy	1 (4%)
Diabetic nephropathy	8 (31%)
Diabetic neuropathy	6 (23%)

Values reported are n (%) for categorical variables and median (interquartile range) for continuous variables

*RAAS* renin–angiotensin–aldosterone system, *ACE* angiotensin converting enzyme, *HDL* high density lipoprotein, *LDL* low density lipoprotein

### Course of HbA1c and body weight

**Fig. 1:**
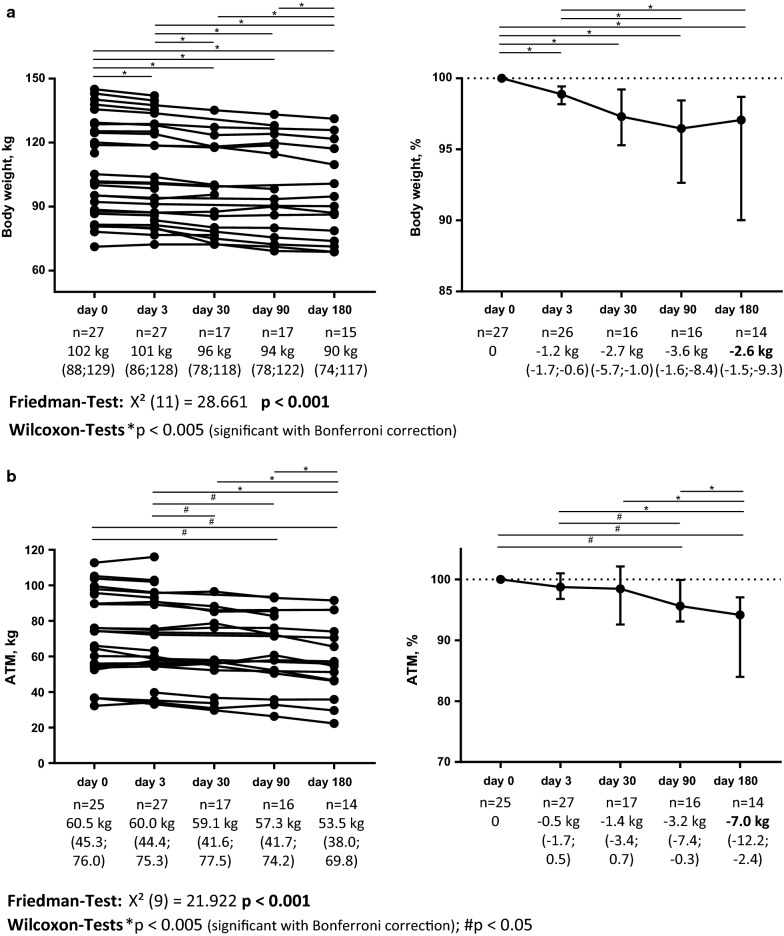
Course of body weight (**a**) and adipose tissue mass (ATM; **b**) under treatment with SGLT2 inhibitors. Left side shows absolute values, right side shows values normalized for baseline value. Whiskers indicate median and interquartile range. Friedman test was performed to test for significant differences during course of follow up; Wilcoxon Signed-Rank test was used to evaluate for differences between respective points of follow up; Bonferroni correction for multiple testing was performed

HbA1c was 9.1% (8.1; 10.9) at baseline and decreased significantly by 0.9% after 30 days and by 0.8% after 6 months after initiation of treatment with a SGLT2 inhibitor, respectively (Additional file 1: Figure S1A). Body weight and BMI at baseline were 101.9 kg (87.8; 128.6) and 36.3 kg/m^2^ (32.6; 46.1), respectively. Decrease of body weight and BMI in this cohort was − 2.6 kg (− 1.5; − 9.3, p = 0.001) and − 0.9 kg/m^2^ (− 0.4; − 3.3, p = 0.001) after 6 months, respectively (Fig. 1a and Additional file 1: Figure S1B).

### Course of body composition and fluid status under treatment with SGLT2 inhibitors

Bioimpedance spectroscopy to assess fluid status and body composition was performed at baseline and after 3, 30, 90 and 180 days of medication with an SGLT2 inhibitor, respectively. ATM was 60.5 kg (45.3; 76.0) at baseline and decreased progressively by − 1.4 kg (− 3.5; 0.7, p = 0.109) at day 30 and − 7.0 kg (− 12.2; − 2.4, p = 0.010) at day 180, compared to baseline, respectively (Fig. 1b). FTI also decreased significantly by − 1.3 kg/m^2^ (− 3.7; − 0.7, p = 0.007) after 6 months (Additional file 1: Figure S1C). There were no significant changes in LTM or LTI during follow up period (Additional file 1: Figure S1D).

**Fig. 2:**
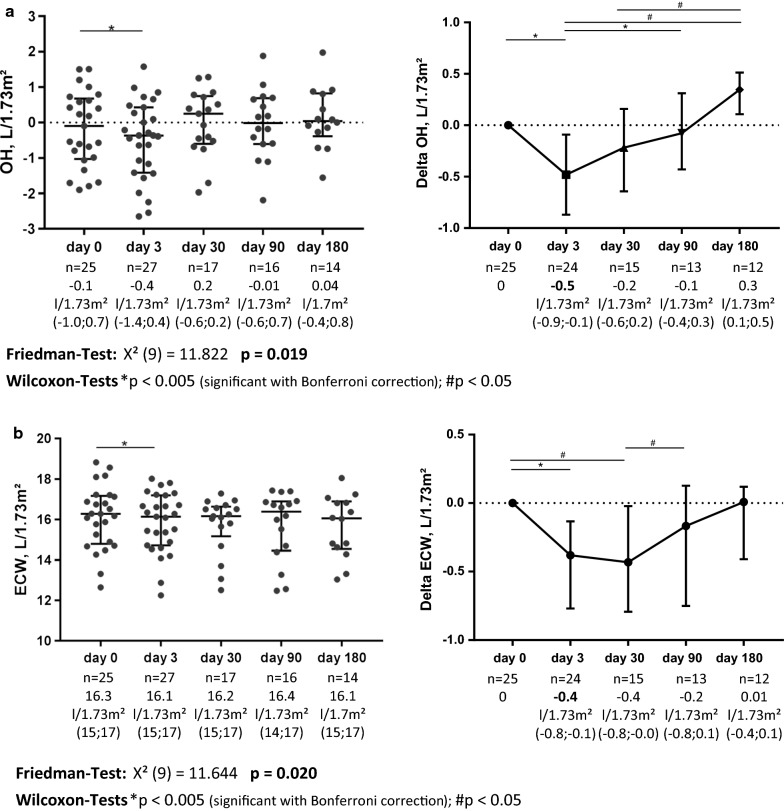
Course of overhydration (OH; **a**) and extracellular water (ECW; **b**) under treatment with SGLT2 inhibitors. Left side shows absolute values, right side shows values normalized for baseline value. Whiskers indicate median and interquartile range. Friedman test was performed to test for significant differences during course of follow up; Wilcoxon Signed-Rank test was used to evaluate for differences between respective points of follow up; Bonferroni correction for multiple testing was performed

Baseline OH and ECW were − 0.1 L/1.73 m^2^ (− 1.0; 0.7) and 16.3 L/1.73 m^2^ (15; 17), respectively. OH and ECW significantly decreased by − 0.5 L/1.73 m^2^ (− 0.1; − 0.9, p = 0.001) and − 0.4 L/1.73 m^2^ (− 0.8; − 1.0, p = 0.002) at day 3 and had returned to the initial value after 3 and 6 months (Fig. 2), excluding a continuous loss of OH or ECW during follow up. Correction for the type of SGLT2 inhibitor in Wilcoxon Signed-Rank tests showed no difference between patients with empagliflozin or dapagliflozin. Course of total body water and intracellular water showed no significant changes during follow up (Additional file 1: Figure S1E, F).

### Course of renin aldosterone axis and blood pressure

**Fig. 3:**
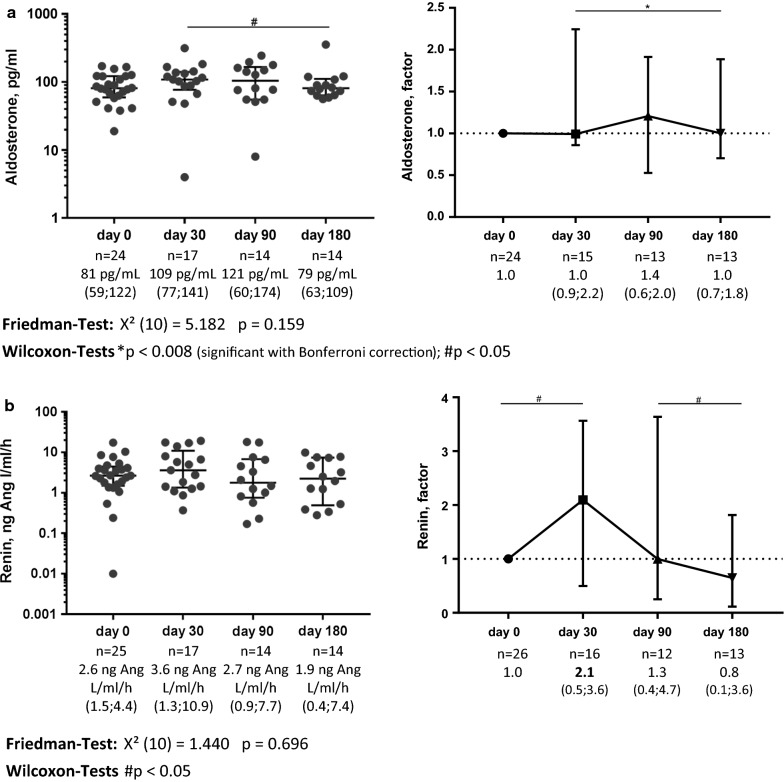
Course of serum aldosterone concentration (**a**) and plasma renin activity (**b**) under treatment with SGLT2 inhibitors. Left side shows absolute values, right side shows values normalized for baseline value. Whiskers indicate median and interquartile range. Friedman test was performed to test for significant differences during course of follow up; Wilcoxon Signed-Rank test was used to evaluate for differences between respective points of follow up; Bonferroni correction for multiple testing was performed

Baseline serum aldosterone concentration and plasma renin activity was 81 pg/mL (59; 122) and 2.6 ng Ang I/mL/h (1.5; 4.4), respectively. During treatment with SGLT2 inhibitors there was a tendency for elevated serum aldosterone concentration and plasma renin activity at day 30, with plasma renin activity being significantly elevated by 2.1-fold (0.5; 3.6, p = 0.044) of baseline value at day 30 (Fig. 3). However, after 6 months plasma renin activity was almost normalized. These changes were observed although nearly all patients had a medication with RAAS inhibitors (Table 1). Baseline plasma NT-pro-BNP was 18 ng/L (18; 73) and showed no significant changes during follow up.

Office systolic and diastolic blood pressure were 141 mmHg (129; 153) and 88 mmHg (76; 99) and both tended to decrease on day 3 (systolic blood pressure − 8 mmHg (− 23; + 6), p = 0.042; diastolic blood pressure − 7 mmHg (− 15; + 4), p = 0.011); there were no significant differences of systolic or diastolic blood pressure after 1, 3 and 6 months, compared to baseline, respectively (Additional file 1: Figure S1G, H). Heart rate showed no significant changes during follow up in this cohort (Additional file 1: Figure S1I).

### Comparison of fluid status under treatment with SGLT2 inhibitors and thiazide diuretics

To further interpret the fluid status of patients treated with SGLT2 inhibitors after 6 months, bioimpedance spectroscopy data was reassessed from a former study of our group involving healthy controls, a comparable group of patients with type 2 diabetes without SGLT2 inhibitor treatment, and patients with hypertension with and without diuretic therapy with hydrochlorothiazide for at least 6 months [24]. Characteristics of these participants are provided in Table 2.

**Table 2:** Characteristics of healthy controls, hypertensive patients with and without hydrochlorothiazide treatment and patients with type 2 diabetes with and without SGLT2 inhibitor treatment

Variable	Healthy	Hypertensive with HCT	Hypertensive without HCT	Type 2 diabetes with SGLT2 inhibitor	Type 2 diabetes without SGLT2 inhibitor	ANOVA p
n	16	14	16	13	5	na
Age (years)	50 (35; 61)	56 (48; 63)	52 (43; 58)	64 (56; 71)	56 (54; 73)	0.0098
Sex (male)	4 (25%)	3 (21%)	5 (31%)	4 (31%)	5 (100%)	0.0472
BMI (kg/m^2^)	29.0 (24.8; 30.4)	30.1 (25.6; 33.4)	28.8 (25.0; 32.5)	32.7 (27.8; 37.6)	32.2 (30.5; 35.6)	0.1195
Body weight (kg)	74.9 (71.1; 84.5)	78.6 (71.0; 100.8)	83.2 (67.4; 95.4)	87.0 (72.6; 113.5)	89.7 (65.8; 98.9)	0.1409
Creatinine (mg/dL)	0.7 (0.6; 0.9)	0.8 (0.7; 0.8)	0.8 (0.6; 1.0)	0.7 (0.5; 0.9)^a^	0.6 (0.6;0.9)	0.5019
Proteinuria (mg/g creatinine)	12.6 (6.3; 16.2)	19.3 (13.5; 24.0)	11.4 (10.2; 18.3)	136 (69;197)^a^	137 (120; 619)	< 0.0001
Renin (ng Ang L/mL/h)	1.3 (0.4; 3.1)	1.8 (0.3; 5.6)	2.8 (0.7; 6.7)	2.5 (0.9; 7.5)	4.5 (2.3; 21)	0.0986
Aldosterone (pg/mL)	161 (123; 194)	106 (75; 163)	102 (96; 146)	79 (64; 105)	112 (68; 147)	0.0254
Medication with RAAS inhibitor	0	11 (79%)	14 (88%)	12 (92%)	5 (100%)	na

Values reported are n (%) for categorical variables and median (interquartile range) for continuous variables. Values reported for patients with type 2 diabetes with SGLT2 inhibitor are values measured at the end of follow up period (day 180 of medication with SGLT2 inhibitor). Hypertensive patients with hydrochlorothiazide treatment included have taken hydrochlorothiazide for at least 6 months

*HCT* hydrochlorothiazide, *OH* overhydration, *ECW* extracellular water, *RAAS* renin–angiotensin–aldosterone system, *na* not applicable

^a^Only baseline value available

**Fig. 4:**
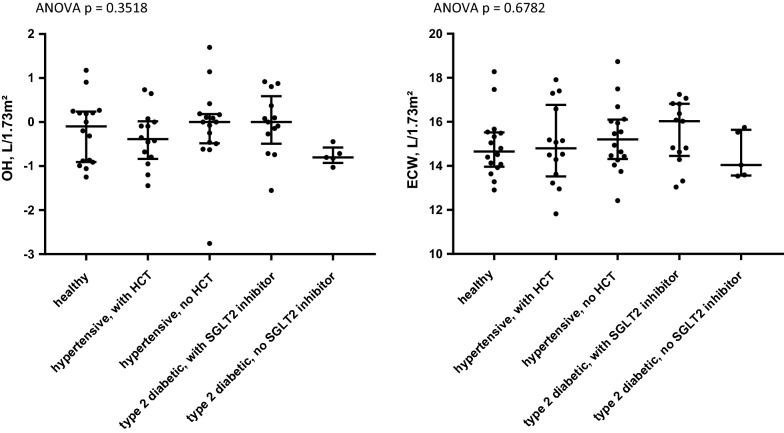
Overhydration (OH; **a**), L/1.73 m^2^ and extracellular water (ECW; **b**), L/1.73 m^2^ in healthy controls, hypertensive patients with and without hydrochlorothiazide and in patients with type 2 diabetes with and without SGLT2 inhibitor. Values reported for patients with type 2 diabetes with SGLT2 inhibitor are values measured at the end of follow up period (day 180 of medication with SGLT2 inhibitor). Hypertensive patients with hydrochlorothiazide (HCT) have taken hydrochlorothiazide for at least 6 months. ANOVA analysis and t-tests performed for each pair reveal no difference in OH or ECW of patients with type 2 diabetes with SGLT2 inhibitors compared to healthy controls, patients with type 2 diabetes without SGLT2 inhibitor and hypertensive patients with and without hydrochlorothiazide. Whiskers indicate median and interquartile range

Patients with type 2 diabetes taking regular medication with SGLT2 inhibitors after 6 months of treatment showed no significant differences in OH or ECW compared to patients with type 2 diabetes without SGLT2 inhibitors (with normal renal function), or to healthy persons (Fig. 4). Patients with hypertension with regular medication with thiazide diuretics also showed no difference in fluid status compared to healthy controls or patients with type 2 diabetes (Fig. 4).

## Discussion

We found that the reduction of body weight during treatment with the SGLT2 inhibitors empagliflozin and dapagliflozin is caused by changes in volume status with decrease of extracellular water during the first days of intake, and by decrease of adipose tissue mass during the following weeks and months. The body weight reduction in our study was − 2.6 kg after 6 months and in the same range as reported previously with a body weight reduction of − 1.9 to − 2.3 after 14 to 24 weeks after initiation of a SGLT2 inhibitor [3, 5, 6, 28].

In our study, the reduction of body weight after 6 months was due to a loss of adipose tissue mass, while lean tissue mass did not change significantly. This is consistent with results from similar studies that used x-ray absorptiometry or calculated indices of adipose tissue mass [5–7]. Other studies involving bioimpedance spectroscopy also found that weight loss under SGLT2 inhibition after 12–104 weeks was mainly via reduction of adipose mass [8–10]. It has been discussed that the loss of adipose mass under SLGT2 inhibition can be attributed to an energy loss due to increased glycosuria [28–30] and increased lipid utilization [31]. Interestingly, besides body fat, epicardial fat was reduced under medication with SGLT2 inhibitors, providing another possible mechanism of reduction of cardiovascular mortality by SGLT2 inhibitors [32, 33].

As the probably most obvious mechanism of cardiovascular risk reduction under SGLT2 inhibition, a diuretic effect as a consequence of increased glucosuria and natriuresis, can be expected [20]. A novel and important finding of the present study is that changes in body fluid status are transient after initiation of SGLT2 inhibitor treatment with empagliflozin or dapagliflozin. Extracellular water was reduced rapidly and significantly after 3 days, but had returned to baseline value when measured after three and 6 months. In a cohort of post-transplant diabetes mellitus, Schwaiger et al. [11] also found a decrease of extracellular fluid after 4 weeks and extracellular water returned to baseline value in the further course. In Japanese patients treated with ipragliflozin, Iizuka et al. [8] found a reduction of total body water by − 0.43 kg at 4 weeks, and a rising tendency at 12 weeks (− 0.37 kg), compared to baseline, respectively, with changes of body fluid mainly caused by extracellular water and no significant changes of intracellular water; however, changes of volume status during the first days of SGLT2 inhibition were not measured and follow up period was shorter, thus missing data on initial as well as long term changes in body fluid status. In our cohort, extracellular water was consistently decreased after 1 and 3 months (− 0.4 L/1.73 m^2^ at day 30 and − 0.2 L/1.73 m^2^ at day 90, compared to baseline, respectively), and showed a return to initial value in the long term follow up (− 0.01 L/1.73 m^2^ at day 180 compared to baseline).

As one consequence of the diuretic effect of SGLT2 inhibitors, a reduction of arterial blood pressure can be expected. In our cohort, along with the course of OH and ECW, systolic and diastolic office blood pressure tended to decrease after 3 days, but there were no significant differences after 3 and 6 months, compared to baseline, respectively. However, in our study, blood pressure was measured only as a single office blood pressure. In studies that considered ambulatory blood pressure monitoring, SGLT2 inhibitors have been shown to reduce blood pressure measured after 3 and 6 months of intake [34–37].

In accordance to our finding of transient decreased OH and ECW, increase of urine volume after initiation of SGLT2 inhibitors has also been found to be transient and was caused rather by natriuresis than by osmotic diuresis due to glycosuria [29, 38]. After initiation of SGLT2 inhibition, an initial, but no long-term elevated natriuresis has been shown, and compensatory mechanisms such as increased sodium reuptake through following tubular transporters and activation of RAAS, have been investigated [38, 39]. Systemic RAAS has been shown to be activated transiently in patients with type 2 diabetes after beginning of SGLT2 inhibitors [39], whereas intrarenal RAAS is not activated after SGLT2 inhibition [40]. SGLT2 inhibitors have been recommended as combination therapy to RAAS inhibitors, especially in diabetic kidney disease [41]. In our study, despite preexisting medication with RAAS inhibitors in nearly all patients, changes of fluid status were accompanied by a trend towards elevated plasma renin activity and serum aldosterone concentration after 30 days, suggesting an increased activity of RAAS, with normalization after 6 months. Our results therefore confirm active counteracting mechanisms of fluid regulation after inhibition of SGLT2 as discussed previously [39].

SGLT2 inhibitors have been suspected to promote the occurrence of stroke due to volume depletion [42, 43], with the Kaplan–Meier curves suggesting elevated risk of stroke during the first time after initiation of SGLT2 inhibition [44]. In the long term, however, stroke incidence is not elevated paralleling the normalization of volume depletion [45]. Another concern about the diuretic effect of SGLT2 inhibitors is that they could promote renal failure. Indeed, parallel to the reduction of ECW after 3 days and 1 month in our cohort, a decline of GFR was observed after initiation of SGLT2 inhibitors [46]. However, in the long term course, SGLT2 inhibitors have even been associated with a slower progression of chronic kidney disease [30, 46]. In our cohort, ongoing fluid loss or reduction of extracellular water as a risk of stroke or prerenal kidney injury was not observed with SGLT2 inhibition, supporting the safety of prescribing SGLT2 inhibitors, as long as counter-regulating mechanisms of fluid status are operative.

At baseline, participants of our study were normally hydrated as both, OH and ECW were in reference ranges. Comparison to a group of hypertensive patients with medication with hydrochlorothiazide as a diuretic agent showed no differences in fluid status of patients with chronic intake of SGLT2 inhibitors, hydrochlorothiazide, or no diuretic medication. This suggests that there is also no ongoing loss of extracellular water under treatment with the diuretic hydrochlorothiazide similar to SGLT2 inhibitors due to counter-regulation. In contrast to our study cohort, patients with heart failure are at risk for fluid overload and extracellular water accumulation, leading to hydropic decompensation and hospitalization [47]. Treatment with SGLT2 inhibitors has been shown to reduce this risk [12] and was found to be safe and efficient in patients with type 2 diabetes and different stages of cardiovascular disease [48]. SGLT2 inhibition could lead to a sustained correction of fluid accumulation in patients starting with an elevated level of overhydration and extracellular water. This is supported by the finding that patients at risk for heart failure, who were treated with empagliflozin, had a reduced need for loop diuretics [14]. In a cohort of chronic kidney disease patients with fluid retention, decrease of extracellular fluid under medication with dapagliflozin was smaller than under medication with furosemide, but larger than under medication with tolvaptan [49]. Our data therefore are compatible with the notion that the protective effect of SGLT2 inhibitors from heart failure is related to their effect on extracellular volume and overhydration. This effect could even be enhanced in patients with overhydration at baseline.

Overall, the diuretic effect of SGLT2 inhibitors seems to be most effective during the initial period of SGLT2 inhibition [38], and we showed that it causes changes in fluid status and could therefore be responsible for fast acting beneficial effects on heart failure; reduction of adipose tissue that we confirmed in the follow up period of SGLT2 inhibition indicates that additional mechanisms for risk reduction in heart failure could gain importance in the long term course, such as lipid utilization [31], reduction of epicardial fat [32, 33], and effects on vascular endothelial function [50].

The limitations of this study are the small number of patients and healthy participants without heart failure or proteinuria, conditions known to be associated with overhydration and edema. The strengths of the study are the direct measurements with the output of quantitative data of volume status and body composition by using bioimpedance spectroscopy. To our knowledge, it is the first study concentrating on the changes of fluid status and body composition during the first days after initiation of SGLT2 inhibitor treatment and during a follow up period of 6 months. Even in our small cohort, significant changes in the intraindividual course of the parameters of interest could be detected, indicating robustness of the observed changes.

## Conclusions

In conclusion, our study shows that body weight reduction under the intake of SGLT2 inhibitors is caused by a decrease of adipose tissue mass after 6 months of intake and by a transient decrease of extracellular water after 3 days and 1 month, which is counterbalanced by upregulation of renin–angiotensin–aldosterone system. Our data argue against an ongoing fluid loss under treatment with SGLT2 inhibitors, which is also not seen in chronic thiazide treatment.

## Additional file

**Additional file 1: Figure S1.** Course of HbA1c (A), BMI (B), fat tissue index (FTI, C), lean tissue index (LTI, D), total body water (E), intracellular water (F), systolic and diastolic blood pressure (G and H) and heart rate (I) under treatment with SGLT2 inhibitors. Left side shows absolute values, right side shows values normalized for baseline value. Whiskers indicate median and interquartile range. Friedman test was performed to test for significant differences during course of follow up; Wilcoxon Signed-Rank test was used to evaluate for differences between respective points of follow up; Bonferroni correction for multiple testing was performed.

## Abbreviations

ATM: adipose tissue mass
BCM: body composition monitor (Fresenius Medical Care)
BMI: body mass index
ECW: extracellular water
FTI: fat tissue index
GFR: glomerular filtration rate
ICW: intracellular water
LTI: lean tissue index
LTM: lean tissue mass
OH: overhydration
RAAS: renin angiotensin aldosterone system
SGLT2: sodium-coupled glucose transporter 2
TBW: total body water
