# Supplementary material for: Effect of SGLT2 inhibitors on body composition, fluid status and renin–angiotensin–aldosterone system in type 2 diabetes: a prospective study using bioimpedance spectroscopy
Source: Cardiovasc Diabetol. 2019 Apr 5;18:46. doi: 10.1186/s12933-019-0852-y (PMC6451223; doi:10.1186/s12933-019-0852-y)
Supplement: Supplementary file 1 — Additional file 1: Figure S1. Course of HbA1c (A), BMI (B), fat tissue index (FTI, C), lean tissue index (LTI, D), total body water (E), intracellular water (F), systolic and diastolic blood pressure (G and H) and heart rate (I) under treatment with SGLT2 inhibitors. Left side shows absolute values, right side shows values normalized for baseline value. Whiskers indicate median and interquartile range. Friedman test was performed to test for significant differences during course of follow up; Wilcoxon Signed-Rank test was used to evaluate for differences between respective points of follow up; Bonferroni correction for multiple testing was performed. [file 12933_2019_852_MOESM1_ESM.pptx]

## Slide 1
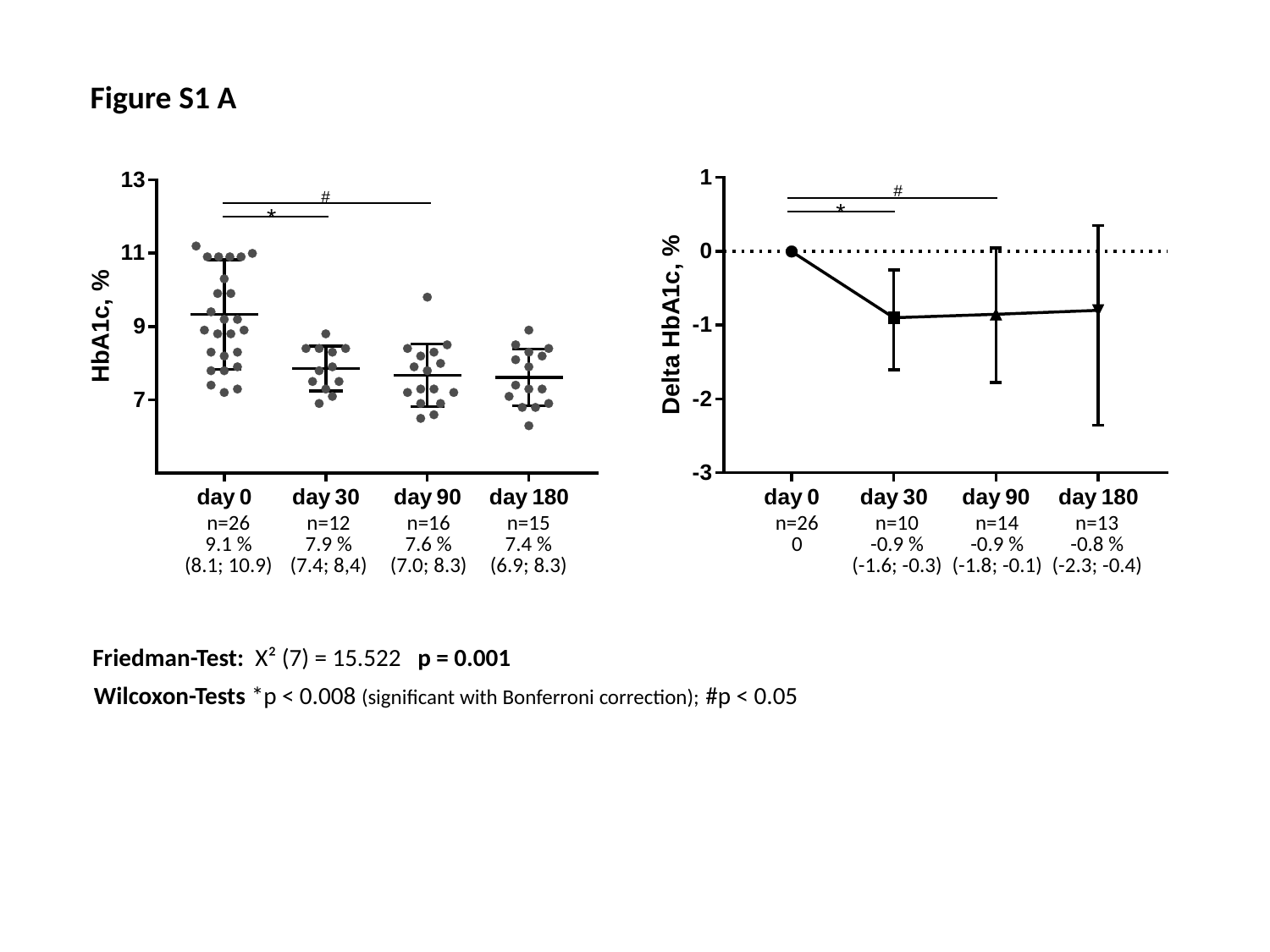

Figure S1 A
| n=26 9.1 % (8.1; 10.9) | n=12 7.9 % (7.4; 8,4) | n=16 7.6 % (7.0; 8.3) | n=15 7.4 % (6.9; 8.3) |
| --- | --- | --- | --- |
| n=26 0 | n=10 -0.9 % (-1.6; -0.3) | n=14 -0.9 % (-1.8; -0.1) | n=13 -0.8 % (-2.3; -0.4) |
| --- | --- | --- | --- |
Friedman-Test: Χ² (7) = 15.522 p = 0.001
Wilcoxon-Tests *p < 0.008 (significant with Bonferroni correction); #p < 0.05

## Slide 2
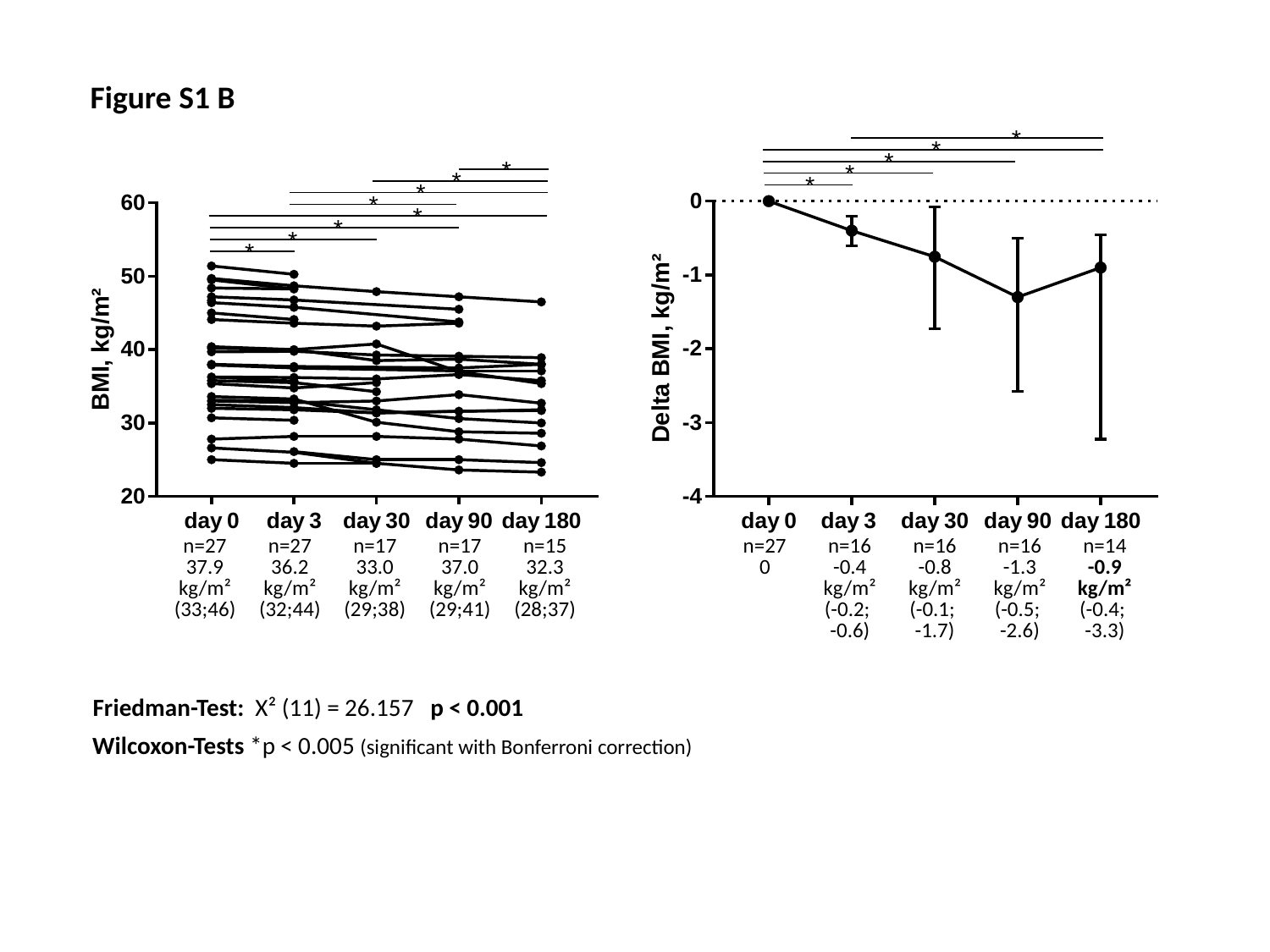

Figure S1 B
| n=27 37.9 kg/m² (33;46) | n=27 36.2 kg/m² (32;44) | n=17 33.0 kg/m² (29;38) | n=17 37.0 kg/m² (29;41) | n=15 32.3 kg/m² (28;37) |
| --- | --- | --- | --- | --- |
| n=27 0 | n=16 -0.4 kg/m² (-0.2; -0.6) | n=16 -0.8 kg/m² (-0.1; -1.7) | n=16 -1.3 kg/m² (-0.5; -2.6) | n=14 -0.9 kg/m² (-0.4; -3.3) |
| --- | --- | --- | --- | --- |
Friedman-Test: Χ² (11) = 26.157 p < 0.001
Wilcoxon-Tests *p < 0.005 (significant with Bonferroni correction)

## Slide 3
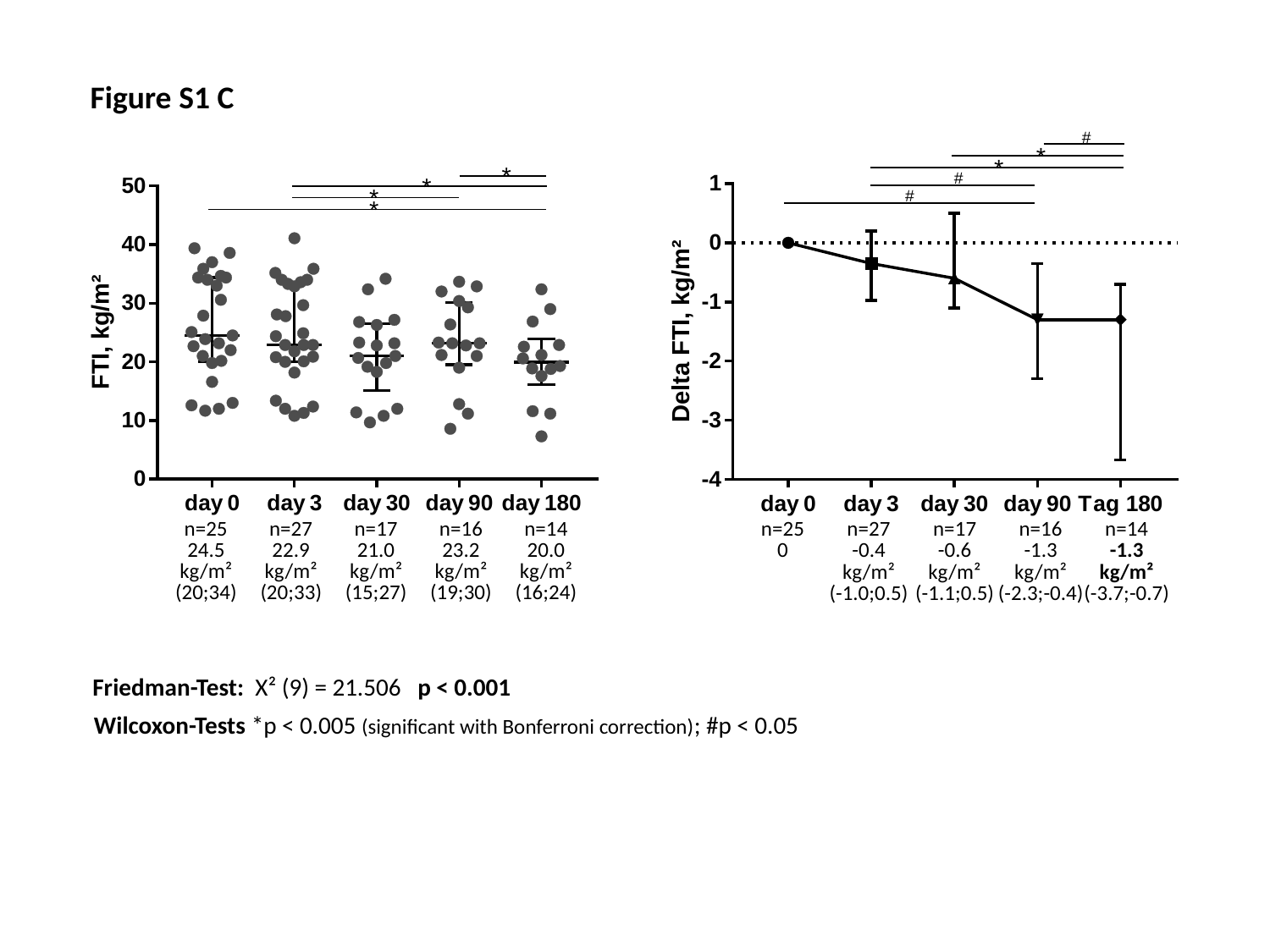

Figure S1 C
| n=25 24.5 kg/m² (20;34) | n=27 22.9 kg/m² (20;33) | n=17 21.0 kg/m² (15;27) | n=16 23.2 kg/m² (19;30) | n=14 20.0 kg/m² (16;24) |
| --- | --- | --- | --- | --- |
| n=25 0 | n=27 -0.4 kg/m² (-1.0;0.5) | n=17 -0.6 kg/m² (-1.1;0.5) | n=16 -1.3 kg/m² (-2.3;-0.4) | n=14 -1.3 kg/m² (-3.7;-0.7) |
| --- | --- | --- | --- | --- |
Friedman-Test: Χ² (9) = 21.506 p < 0.001
Wilcoxon-Tests *p < 0.005 (significant with Bonferroni correction); #p < 0.05

## Slide 4
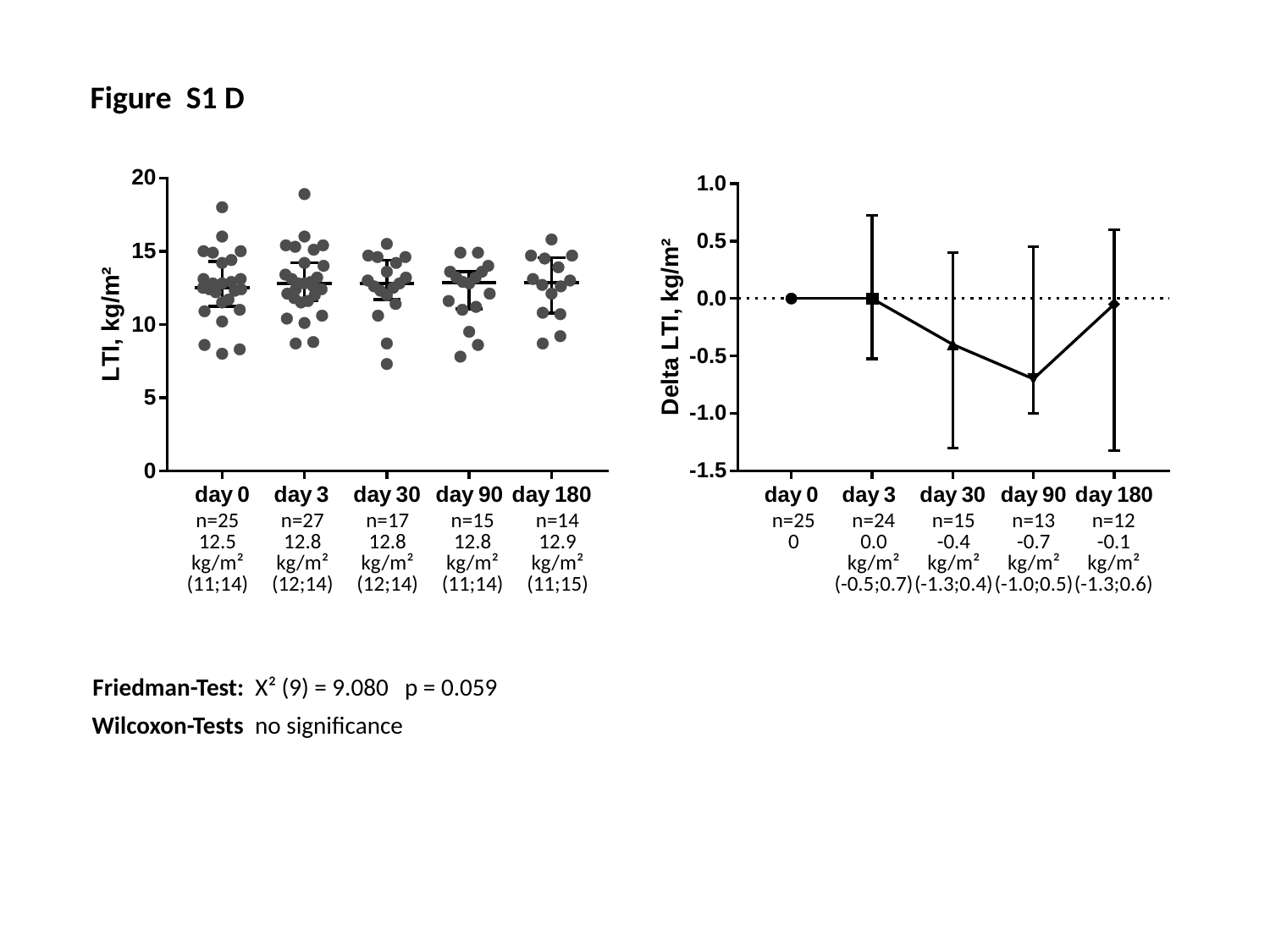

Figure S1 D
| n=25 12.5 kg/m² (11;14) | n=27 12.8 kg/m² (12;14) | n=17 12.8 kg/m² (12;14) | n=15 12.8 kg/m² (11;14) | n=14 12.9 kg/m² (11;15) |
| --- | --- | --- | --- | --- |
| n=25 0 | n=24 0.0 kg/m² (-0.5;0.7) | n=15 -0.4 kg/m² (-1.3;0.4) | n=13 -0.7 kg/m² (-1.0;0.5) | n=12 -0.1 kg/m² (-1.3;0.6) |
| --- | --- | --- | --- | --- |
Friedman-Test: Χ² (9) = 9.080 p = 0.059
Wilcoxon-Tests no significance

## Slide 5
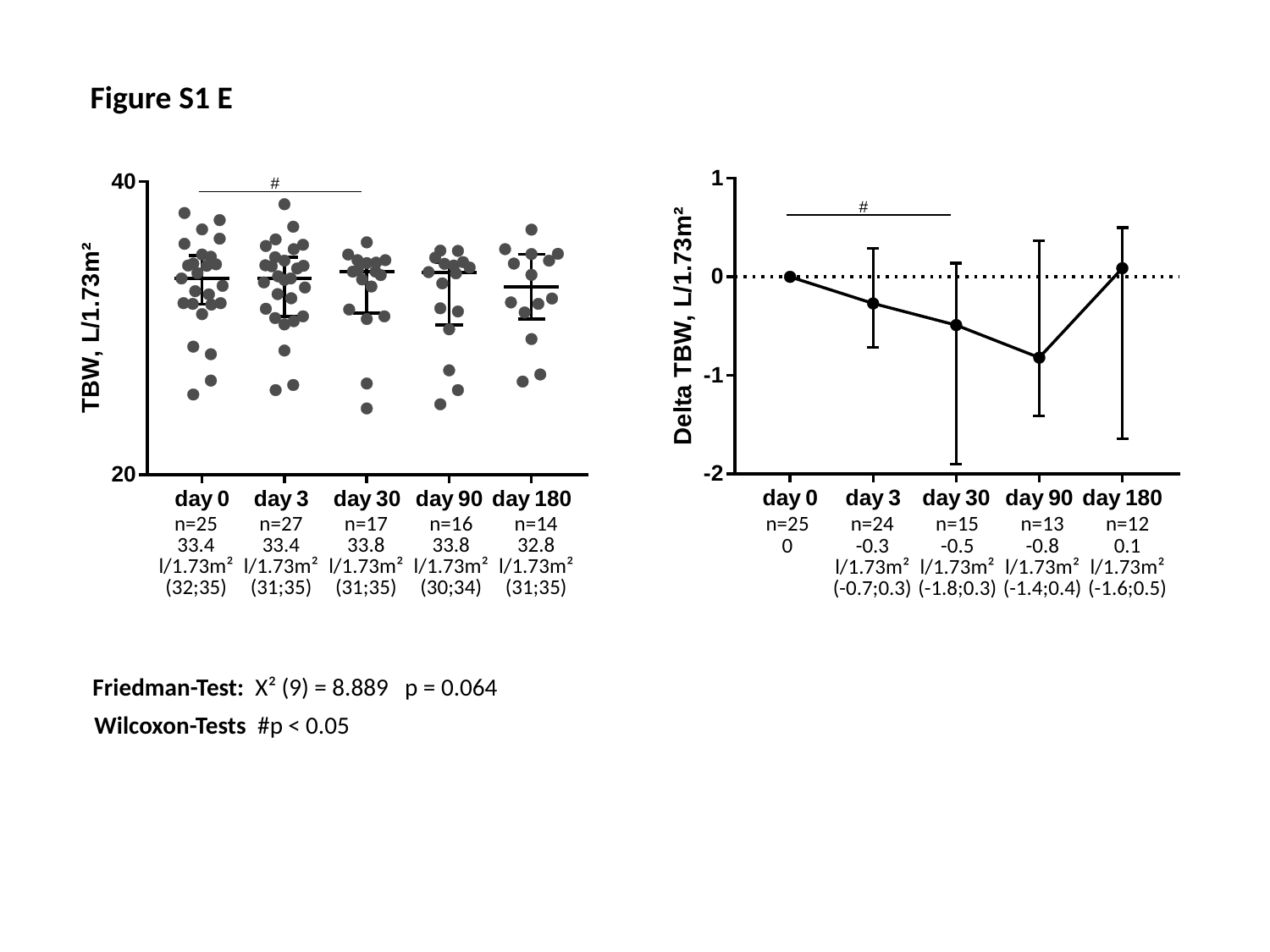

Figure S1 E
| n=25 33.4 l/1.73m² (32;35) | n=27 33.4 l/1.73m² (31;35) | n=17 33.8 l/1.73m² (31;35) | n=16 33.8 l/1.73m² (30;34) | n=14 32.8 l/1.73m² (31;35) |
| --- | --- | --- | --- | --- |
| n=25 0 | n=24 -0.3 l/1.73m² (-0.7;0.3) | n=15 -0.5 l/1.73m² (-1.8;0.3) | n=13 -0.8 l/1.73m² (-1.4;0.4) | n=12 0.1 l/1.73m² (-1.6;0.5) |
| --- | --- | --- | --- | --- |
Friedman-Test: Χ² (9) = 8.889 p = 0.064
Wilcoxon-Tests #p < 0.05

## Slide 6
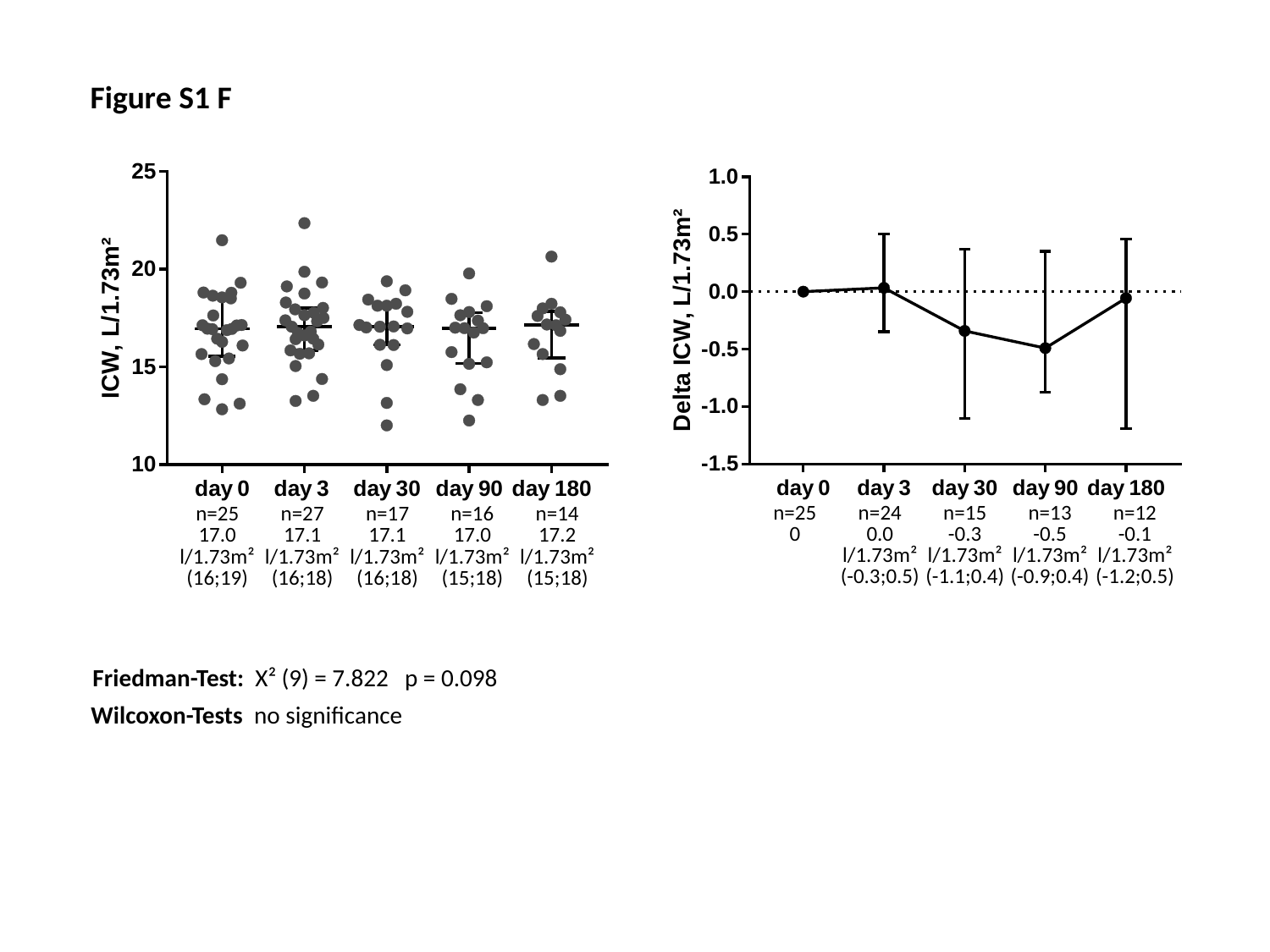

Figure S1 F
| n=25 0 | n=24 0.0 l/1.73m² (-0.3;0.5) | n=15 -0.3 l/1.73m² (-1.1;0.4) | n=13 -0.5 l/1.73m² (-0.9;0.4) | n=12 -0.1 l/1.73m² (-1.2;0.5) |
| --- | --- | --- | --- | --- |
| n=25 17.0 l/1.73m² (16;19) | n=27 17.1 l/1.73m² (16;18) | n=17 17.1 l/1.73m² (16;18) | n=16 17.0 l/1.73m² (15;18) | n=14 17.2 l/1.73m² (15;18) |
| --- | --- | --- | --- | --- |
Friedman-Test: Χ² (9) = 7.822 p = 0.098
Wilcoxon-Tests no significance

## Slide 7
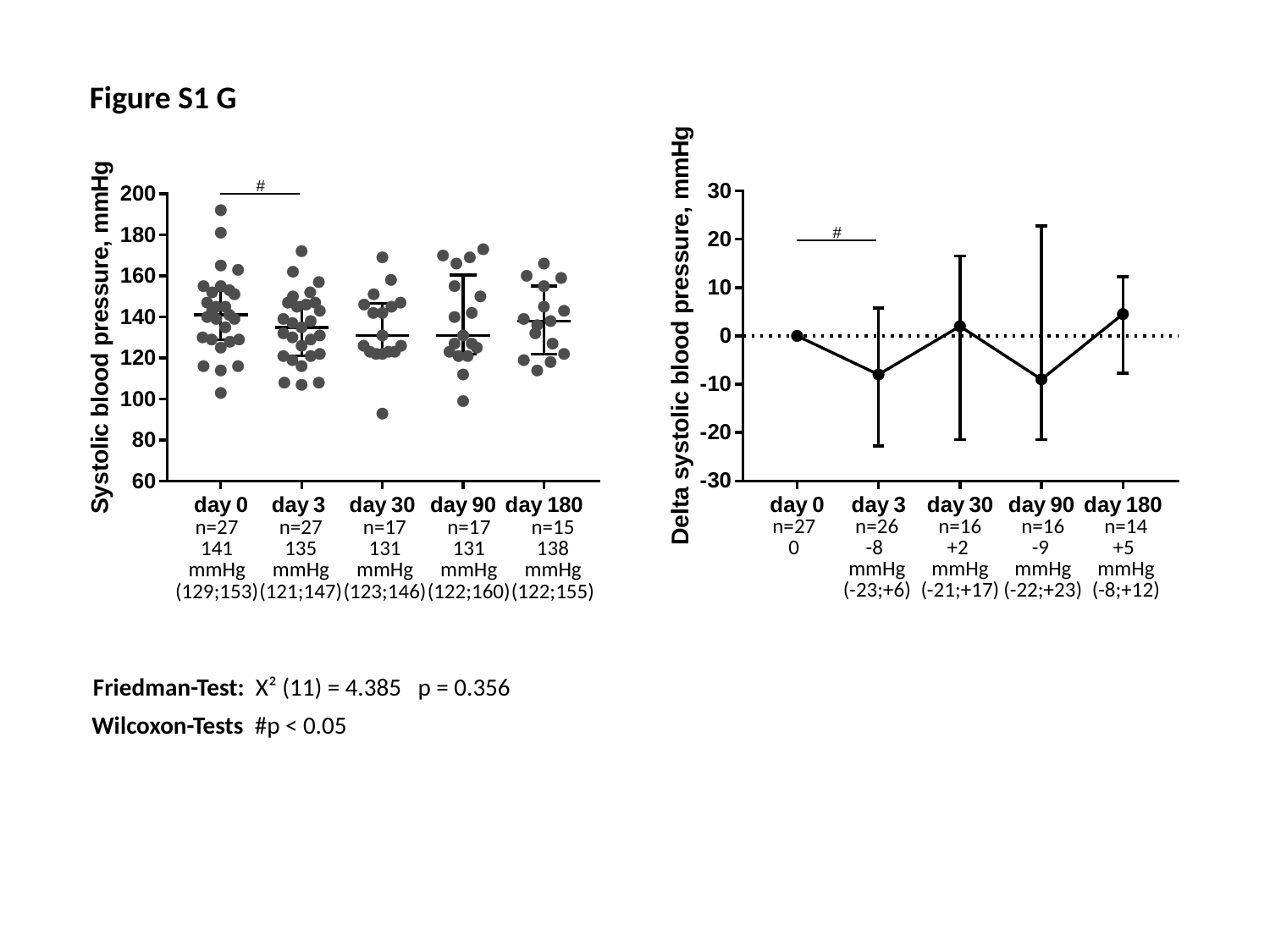

Figure S1 G
| n=27 0 | n=26 -8 mmHg (-23;+6) | n=16 +2 mmHg (-21;+17) | n=16 -9 mmHg (-22;+23) | n=14 +5 mmHg (-8;+12) |
| --- | --- | --- | --- | --- |
| n=27 141 mmHg (129;153) | n=27 135 mmHg (121;147) | n=17 131 mmHg (123;146) | n=17 131 mmHg (122;160) | n=15 138 mmHg (122;155) |
| --- | --- | --- | --- | --- |
Friedman-Test: Χ² (11) = 4.385 p = 0.356
Wilcoxon-Tests #p < 0.05

## Slide 8
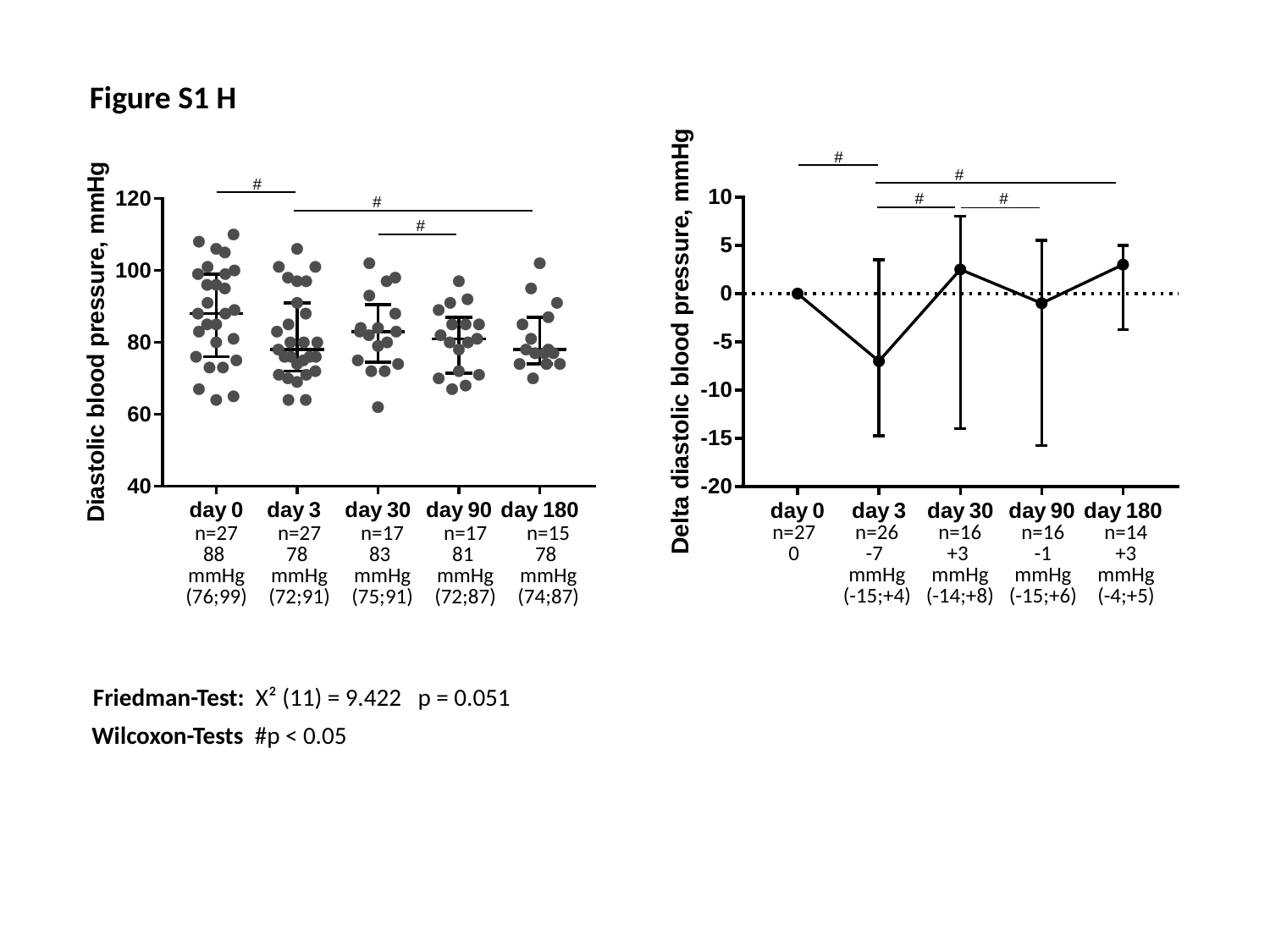

Figure S1 H
| n=27 0 | n=26 -7 mmHg (-15;+4) | n=16 +3 mmHg (-14;+8) | n=16 -1 mmHg (-15;+6) | n=14 +3 mmHg (-4;+5) |
| --- | --- | --- | --- | --- |
| n=27 88 mmHg (76;99) | n=27 78 mmHg (72;91) | n=17 83 mmHg (75;91) | n=17 81 mmHg (72;87) | n=15 78 mmHg (74;87) |
| --- | --- | --- | --- | --- |
Friedman-Test: Χ² (11) = 9.422 p = 0.051
Wilcoxon-Tests #p < 0.05

## Slide 9
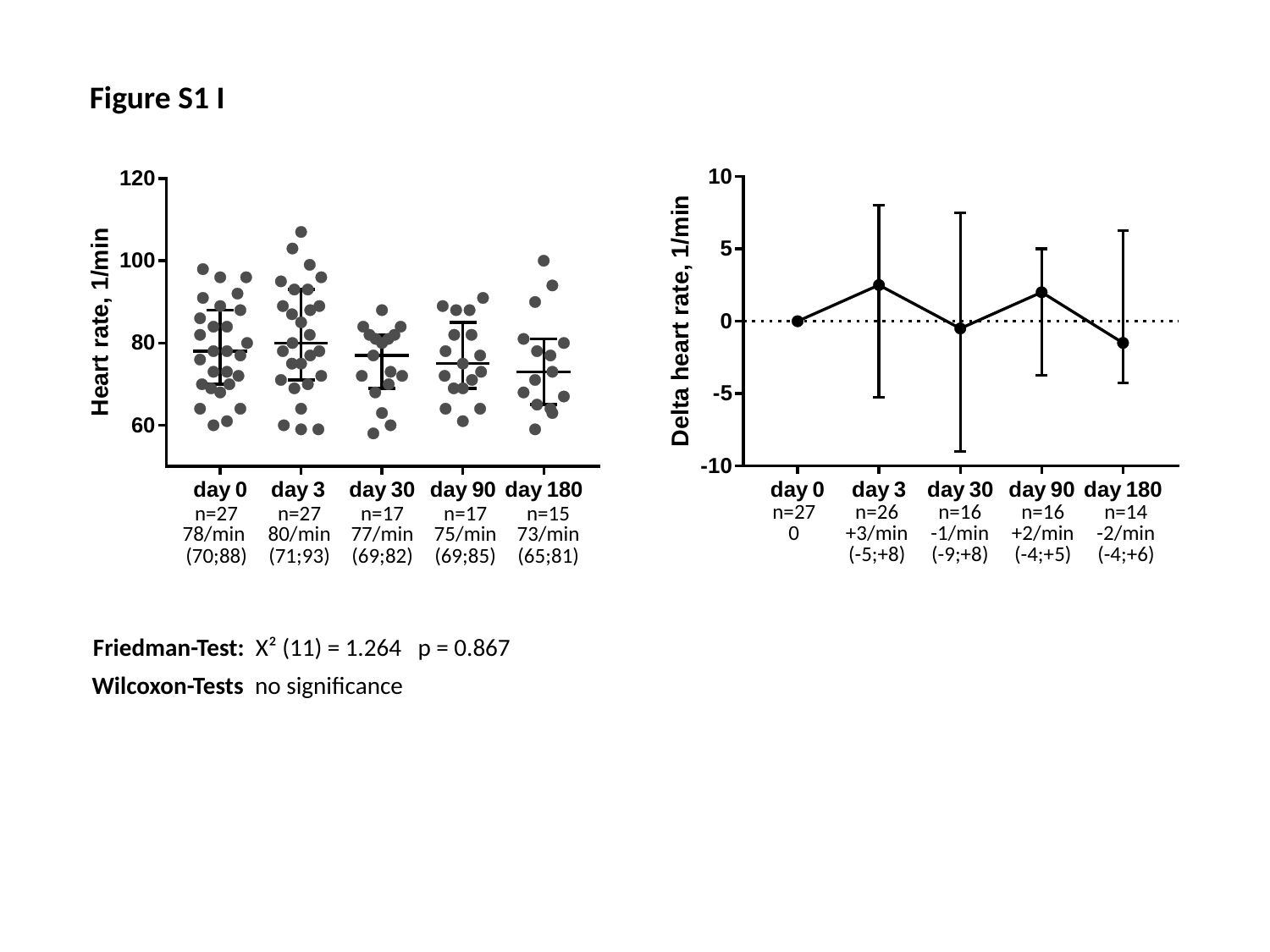

Figure S1 I
| n=27 0 | n=26 +3/min (-5;+8) | n=16 -1/min (-9;+8) | n=16 +2/min (-4;+5) | n=14 -2/min (-4;+6) |
| --- | --- | --- | --- | --- |
| n=27 78/min (70;88) | n=27 80/min (71;93) | n=17 77/min (69;82) | n=17 75/min (69;85) | n=15 73/min (65;81) |
| --- | --- | --- | --- | --- |
Friedman-Test: Χ² (11) = 1.264 p = 0.867
Wilcoxon-Tests no significance
